# Exploring the Metabolome and Antimicrobial Properties of *Capsicum annuum* L. (Baklouti and Paprika) Dried Powders from Tunisia

**DOI:** 10.3390/molecules29225236

**Published:** 2024-11-05

**Authors:** Annalisa Serio, Francesca Maggio, Anis Ben Hsouna, Rania Ben Saad, Cosimo Taiti, Stefania Garzoli

**Affiliations:** 1 Department of Bioscience and Technology for Food, Agriculture and Environment, University of Teramo, 64100 Teramo, Italy; aserio@unite.it (A.S.); fmaggio@unite.it (F.M.); 2 Department of Environmental Sciences and Nutrition, Higher Institute of Applied Sciences and Technology of Mahdia, University of Monastir, Monastir 5000, Tunisia; benhsounanis@gmail.com; 3 Laboratory of Biotechnology and Plant Improvement, Centre of Biotechnology of Sfax, University of Sfax, Sfax 3018, Tunisia; raniabensaad@gmail.com; 4 Department of Agri-Food and Environmental Science, Università di Firenze, 50019 Firenze, Italy; cosimo.taiti@unifi.it; 5 Department of Drug Chemistry and Technology, Sapienza University, 00185 Rome, Italy

**Keywords:** HS-SPME-GC/MS analysis, PTR-ToF-MS analysis, secondary metabolites, antimicrobial activity

## Abstract

In this study, for the first time, the volatile fraction from two domesticated *Capsicum annuum* accessions (“Paprika” and “Baklouti”) collected in Tunisia was investigated by two complementary analytical techniques, such as Solid-Phase Microextraction–Gas Chromatography/Mass Spectrometry (SPME-GC/MS) and Proton Transfer Reaction–Time-of-Flight–Mass Spectrometry (PTR-ToF-MS). The obtained results highlighted the presence of a high number of Volatile Organic Compounds (VOCs), including monoterpene and sesquiterpene compounds with *α*-curcumene, I-zingiberene, *β*-bisabolene and *β*-sesquiphellandrene as the major components. In addition, GC/MS was used to investigate the non-volatile chemical composition of the dried powders and their extracts, which were found to be rich in sulfur compounds, fatty acids and sugars. Eleven bacterial strains were chosen to assess the antimicrobial effectiveness of the extracts. The results showed that the extracts exhibited strain-dependent behavior, and the type strains displayed a greater susceptibility to the treatments, if compared to the wild strains, and, in particular, showed the best antimicrobial activity against Gram-positive bacteria, such as *Listeria monocytogenes* and *Staphylococcus aureus.*

## 1. Introduction

The food industry is facing great challenges nowadays in developing natural antimicrobials and antioxidants for foods, responding to the growing consumer demand for safe, healthy and green foods, reducing the use of synthetic chemical preservatives. Food safety is an essential aspect to counteract the high incidence rates of foodborne diseases caused by pathogenic strains, including *Salmonella*, *Escherichia coli*, and *Listeria monocytogenes,* linked to the emergence of multidrug resistance to conventional antimicrobial agents [1]. Used as bio-preservatives and/or flavoring agents to improve the organoleptic properties of food products, herbs and spices are considered outstanding antioxidant and antimicrobial agents. This is the case of Mentha, Coriandrum, Cuminum, Capsicum and Foeniculum seeds, plants and spices [2,3], frequently used in the formulation of traditional products in the Mediterranean region and North African countries [4,5]. Based on toxicological research, the main phytochemical compounds of traditional spices and herbs are considered toxicologically safe [2]. In addition, several studies have reported a high correlation between antioxidant activity, antimicrobial efficacy and the level of bioactive components present in some herbs and spice preparations [3,4]. Generally, crude extracts of herbs and spices consist of a large pool of phytochemicals, with the main bioactive compounds constituting up to 85%, while other components are found at trace levels [5]. Extracts and essential oils of Capsicum (*Capsicum annuum* L.), belonging to the Solanaceae family, have also been used as a traditional medicinal alternative and are commonly recommended as antioxidant, antiseptic, antimicrobial, antifungal, aperitif, digestive, antispasmodic, anti-inflammatory and diuretic agents [6].

*Capsicum annuum* L., a flowering plant, is widely cultivated for its hot and sweet chili peppers of its thousands of varieties and cultivar. The genus *Capsicum* includes over thirty species, five of which (*C. annuum, C. frutescens, C. chinense, C. baccatum*, and *C. pubescens*) are domesticated and mainly grown for consumption [7]. The fruits of this species are common ingredients in the cuisines of many countries around the world and can also be grown for ornamental purposes or for medicinal uses [8]. Tunisian peppers are large, slightly curved pods, averaging 15–20 cm (6–8 inches) long, and have a conical shape that tapers to a pointed tip at the stemless end. The skin is shiny, waxy, hard and wrinkled and ripens from green to bright red. Beneath the surface, the flesh is thin, crisp, watery and pale red, enclosing a central cavity filled with small, cream-colored seeds. In particular, Paprika has a sweet flavor, while Baklouti, a pepper native to Tunisia in North Africa, has a unique and slightly spicy flavor. The peppers are available from summer to fall and are an excellent source of vitamin C, folate, vitamins A, B6 and E and potassium. These fruits are suitable for both raw and cooked applications such as roasting or stir-frying. The peppers can be sliced and tossed in salads, chopped into sauces and gravies, infused in oils for added flavor or dried and ground into a powder, and they are widely used to season and flavor various foods. Baklouti is a cultivar of chili pepper of the *Capsicum annuum* species native to Tunisia and it is and is very popular with consumers as it is a medium-hot chili.

In this study, for the first time, two *Capsicum* accessions, Paprika and Baklouti, from Tunisia, were chemically investigated using a metabolomic approach based on two complementary techniques, such as Headspace Solid-Phase Microextraction–Gas Chromatography/Mass Spectrometry (HS-SPME-GC/MS) and Proton Transfer Reaction–Time-of-Flight–Mass Spectrometry (PTR-ToF-MS), to describe their volatile fraction. Further, GC/MS analysis on dry powders and extracts, after derivatization, was carried out to examine the non-volatile content. Both spices were also tested to evaluate their antibacterial efficacy against a panel of Gram-positive and Gram-negative bacteria, showing a selective response against the tested pathogens. The obtained results suggest that the Paprika and Baklouti peppers, thanks to their numerous bioactive secondary metabolites, could contribute to the control of microbial growth in different food products.

## 2. Results and Discussion

### 2.1. SPME-GC/MS: Chemical Volatile Composition of the Spices

Aroma is an important characteristic of the flavor and quality of pepper fruit and is closely related to the VOCs present in the sample [9,10]. However, as reported by a previous study conducted on 147 samples of dried peppers, the compositional profile (variability between components and their levels) can vary significantly among different *Capsicum* varieties [11]. In fact, the VOC content was investigated by Heng et al. [12] in two pepper varieties, *C. chinenses* and *C. annuum,* by using the HS-SPME technique. Twenty-nine VOCs were identified, which were mainly esters, aldehydes, alcohols and hydrocarbons, but with *C. chinenses* having much higher VOC levels. In another work, a gas chromatography–olfactometry–mass spectrometry analysis was performed on *C. chinense* ‘JT-1′ pepper fruits harvested from 1 to 7 weeks after flowering. The results of the investigation revealed a variable concentration of VOCs depending on the different harvest periods [13]. The VOC levels in fresh *C. annuum* spices, chili flakes, and industrial and traditional spices were analyzed by SPME-GC/MS. The results demonstrated that thermal process, moisture content and time are critical parameters in determining the VOC profile during the production stage of pepper [14]. In addition, the geographical origin, cultivar and ripening process also influence pepper aroma type [15]. Generally, the main VOCs detected in pepper are esters and terpenes. Other compounds present in trace amounts include phenolic derivatives, alcohols and other hydrocarbons and nitrogen–sulfur compounds [16,17]. Pepper is known to be a natural source of carotenoids, and most of the works in the literature investigate this chemical class of compounds [18,19]. A recent study described the content in terms of carotenoids, phenolic compounds, steroids and capsaicinoids in the hydro-methanolic seed extract of *C. annuum* from Tunisia [20]; nevertheless, on the other hand, data on the volatile fraction are more scarce. In the present work, we aimed to provide more information on the less investigated volatile composition of this matrix. In particular, to the best of our knowledge, the present study is the first to use SPME-GC/MS and PTR-ToF-MS techniques to determine the volatilome of *Capsicum annum* L. collected in Tunisia.

The analyses conducted by SPME-GC/MS allowed us to identify 31 Volatile Organic Compounds (VOCs), listed in Table 1.

The obtained results highlighted, for the two spices, an almost overlapping chemical composition from a qualitative point of view. Both peppers were rich in sequiterpene compounds, which represented 65.4% (Paprika) and 65.4% (Baklouti) of the total composition. As regards Paprika pepper, its compositional profile was predominantly characterized by *α*-curcumene (16.0%), *β*-sesquiphellandrene (14.7%) and *β*-bisabolene (13.0%). On the other hand, Baklouti showed I-zingiberene (25.5%) as the most abundant component followed by *α*-curcumene (19.7%), *β*-sesquiphellandrene (13.8%) and *β*-bisabolene (10.7%). In addition to I-zingiberene, for some compounds such as limonene, linalool, (-)-carvone and *β*-caryophyllene, the quantitative differences detectable between the two spices were more evident. Other minor compounds, whose relative quantities ranged from 0.1 to 0.9%, such as camphor, *α*-limonene diepoxide and *trans*-*β*-farnesene, were present in Paprika pepper, while anethole, isobornyl acetate, carvacrol, eugenol, *β*-copaene, trans-geranylacetone, *trans*-calamenene and *α*-calacorene (ranging from 0.1 to 1.0%) were found only in Baklouti pepper.

The sesquiterpene hydrocarbon zingiberene, found in the dried rhizomes of Indonesian ginger, *Zingiber officinalis*, has been shown to have a considerable spectrum of biological activity such as antiviral and antiulcer [21]. On the other hand, curcumene, also a sesquiterpene hydrocarbon and the main metabolite of ginger rhizomes, is known for its insecticidal [22] and antimicrobial properties [23]. These results show that both peppers can be used as a biorational source of zingiberene and curcumene.

Lastly, considering the high content of phytochemicals in the investigated spices, these could also represent a source of bioactive compounds for possible future applications if encapsulated inside specific carriers such as hyper-cross-linked polymers (HCPs) that possess characteristics that also make them usable in the biomedical area as antimicrobials [24].

### 2.2. PTR-ToF-MS: Determination of Volatile Compounds from Spices

PTR-ToF-MS analyses provided a volatile fingerprint from Paprika and Baklouti peppers, with high mass resolution, short acquisition times and no sample modifications. A total of 56 different peak signals were detected from the two peppers studied. By using the same technique, 101 volatile compounds were identified in six common market spices (black/white pepper, chili paprika, cinnamon, nutmeg and saffron) showing a pattern that varied by spice type [25]. As reported in Table 2, the two pepper accessions exhibited a similar volatile profile, consistent with the findings previously reported by Taiti et al. [26] on *C. annuum* dry matrices. However, compared to the previous study, we observed a higher emission of terpene compounds from both Paprika and Baklouti peppers. Indeed, the PTR results revealed a volatile profile characterized by different classes of terpene compounds, including sesquiterpenes (mostly detected at *m*/*z* 205, 123, 109), oxygenated terpenes (mostly detected at *m*/*z* 153, 123, 109), and monoterpenes (mostly detected at *m*/*z* 137, 123, 109). In addition, we observed a greater emission of sulfur compounds (*m*/*z* 49, 63, 111) from Paprika compared to Baklouti. These differences between the two studied accessions highlight a distinct chemical composition and potential flavor contributions of these pepper varieties compared to other *C. annuum* accessions. It is important to note that these compounds have a different odor threshold perception. For example, monoterpenes generally have lower odor thresholds and are often perceived at lower concentrations, contributing significantly to the aroma profile even in small amounts. In contrast, sesquiterpenes typically have higher odor thresholds, requiring higher concentrations to be detected. Sulfur compounds, such as thiols, sulfides, and disulfides, often have extremely low odor thresholds (often pungent odors) and can contribute significantly to the overall aroma of the peppers. These different intensity emissions observed for these compounds can significantly impact the overall aroma profile of the two peppers. So, the detected volatile profile suggests that both Paprika and Baklouti peppers may have a more intense and complex aroma compared to what was previously reported [26], potentially enhancing their sensory appeal and culinary value.

In this work, we have used HS-SPME-GC/MS and PTR-ToF-MS, complementary techniques, because both only require small amounts of sample for analysis without the need for any treatment and with a short extraction time. With PTR-ToF-MS, it is possible to obtain the entire volatile spectrum in real time (including low-molecular-weight compounds) with high sensitivity and high time resolution, although some compounds are ambiguously identified [27,28,29]. On the other hand, the HS-SPME technique is ideal for MS applications as it combines simple and efficient sample preparation with versatile and sensitive detection [30,31]. In a recent study, remarkable results were obtained, applying the same combined approach, for the analysis of eleven coffee husks of Coffea arabica with different primary processing treatments from three latitudes of Yunnan [32]. Therefore, using a combined approach, the present study represents a first step towards a better comprehension of the volatile fraction of the pepper samples [33].

### 2.3. Chemical Composition of the Spices After Derivatization

Direct injection of the silylated samples enabled the identification of fatty acids and sugars (Table 3). In Baklouti pepper, molecules belonging to both chemical classes were detected, while in Paprika, only fatty acids were found. In detail, among sugars and fatty acids, glycerol (54.2%), D-psicofuranose (10.5%) and oleic acid (9.1%) were the most abundant molecules in Baklouti pepper. Many foods can also be sources of glycerin, such as little extras that you add to food, like sauces and dressings. This trihydroxy sugar alcohol is an intermediate in carbohydrate and lipid metabolism and it has been reported to have antibacterial properties [34]. Further, it is a common metabolite in microorganisms and invertebrates with protective functions against anaerobic and osmotic stresses [35]. In Paprika pepper, oleic acid (47.3%) was the major fatty acid followed by linoleic acid (28.1%) and palmitic acid (17.7%). These three fatty acids were also reported as the major ones in all Paprika oils collected in different countries [36]. However, the content of fatty acids and its trend can vary depending on the variety of pepper; in fact, in a previous study, it was reported that in the fruit of spicy pepper, the average amount of fatty derivatives was significantly higher than that of non-spicy pepper [37]. Further, the trend of the percentage values of individual fatty acids can also vary depending on the collection locations and the different production and processing methods; for instance, Zaki et al. [38] reported that, in order, linoleic acid, oleic acid and palmitic acid were the main fatty acids found in paprika powder collected in Morocco.

### 2.4. Chemical Composition of the Extracts After Derivatization

The analyses of the silylated extracts revealed the presence of nineteen compounds, listed in Table 4. The obtained results highlighted the presence of sulfur compounds with a higher relative total percentage in Paprika pepper than Baklouti. Dimethyl sulfone was the main sulfur compound in both spices. This sulfur-tasting compound belongs to the class of organic compounds known as sulfones and it is derived from dietary sources such as garlic [39]. Over time, it has become a popular dietary supplement [40] due in part to its anti-inflammatory effects [41]. In line with the results obtained by the analyses conducted on the dry powders, sugars were present only in Baklouti pepper while fatty acids were detected in both matrices, with a prevalence of oleic acid (46.8% Paprika; 54.5% Baklouti).

### 2.5. Antimicrobial Activity of Plant Extracts Based on Minimum Inhibitory Concentrations and Minimum Bactericidal Concentrations

Table 5 illustrates the Minimum Inhibitory Concentrations (MICs) of extracts, expressed as mg/mL and derived from Baklouti and Paprika, for the strains under investigation, as observed after 24 and 48 h of incubation at 37 °C. As the MIC is considered as the minimum concentration capable of inhibiting microbial growth, lower concentrations indicate a greater effect of the extract and a greater sensitivity of the strain. As expected, Gram-negative bacteria were less susceptible to the extracts, as a consequence of the presence of the outer membrane in the cell wall. Nevertheless, the antimicrobial effectiveness of the extracts exhibited strain-dependent behavior, where the type strains displayed a greater susceptibility to the treatments, if compared to the wild strains. In fact, the MICs for the wild strains frequently exceed the maximum concentration employed during the analysis (50 mg/mL), as shown for *S. enterica* serovar Enteritidis and Veneziana. Conversely, the extracts exhibited notable antimicrobial effectiveness against the type strains, where the MICs ranged from <0.39 and 12.5 mg/mL. The increased range of MICs observed for the wild strains is likely due to the acquisition of resistance in response to the selective pressure of environmental stress induced by antimicrobial treatments implemented in the food supply chain, or by previous contacts with herbs and spices in food products [42]. Considering the strains’ response in the presence of DMSO, Baklouti and Paprika extracts showed the best antimicrobial activity against Gram-positive bacteria, such as *L. monocytogenes* and *S. aureus*, in accordance with previous results [43]. In particular, *S. aureus* ATCC 43300 was inhibited by the Paprika extract employed at the lowest concentration (0.39 mg/mL). The Minimum Bactericidal Concentration (MBC) results (Table 5) corroborated the behavior observed for the MICs after 24 and 48 h. *L. monocytogenes* and *S. aureus* are common foodborne pathogens which can contaminate a wide variety of fresh (milk, seafood, meat and vegetables) and processed food products [44]. Thus, the antimicrobial activity exerted by Baklouti and Paprika extracts against the two pathogens could contribute to the control of the microbial growth in different food products.

The good antimicrobial properties of both extracts can be attributed to the bioactive compounds revealed in the extracts and in the dried powder samples. Sulfur compounds play an important role as an antibacterial agent [45]; in particular, dimethyl sulfone has been shown to have an antibacterial effect against *Escherichia coli* (MDRE21) and *Salmonella enterica* Kinshasa (SK132) [46] as well as against vancomycin-resistant *Enterococcus faecium* [47]. The determined antimicrobial activity may also be influenced by the fatty acid-based chemical composition; in fact, the unsaturated fatty acids have been documented to be strong antimicrobial compounds [48]. Their mechanism of action includes membrane destabilization, increased permeability, disruption of the electron transport chain and inhibition of some membrane-associated enzymes. In particular, unsaturated fatty acids, including oleic and linoleic, significantly inhibit glucosyltransferase (GTase), an essential transmembrane protein that facilitates glucan synthesis in bacterial cells [49]. Other authors also reported the antimicrobial effectiveness of oleic acid against Gram-positive bacteria including *S. aureus* [50]. Finally, monoterpenes and sesquiterpenes detected in the powder peppers exert antibacterial power. In detail, the antioxidant, antimicrobic and antibiofilm properties of carvone, limonene and linalool have been documented by Bouyahya et al. [51] and Tardugno et al. [52] against several bacterial and fungal species, with *S. aureus* and *L. monocytogenes* being the most sensitive. On the other hand, the antibacterial properties of the sesquiterpenes, such as *β*-caryophyllene, zingiberene and α-curcumene, can contribute via the disruption of the cell membranes and the cell walls, thereby influencing the permeability, the release of intracellular components and the intracellular leakage [53,54]. In conclusion, the Baklouti and Paprika extracts were demonstrated to be rich in bioactive compounds, which can exert stressing and antimicrobial effects on bacterial cells.

## 3. Materials and Methods

### 3.1. Plant Material

Tunisian peppers, Baklouti and Paprika, were purchased in 2023 from a local producer specialized in the production of traditional products located in SfaxTunis, Tunisia. The peppers were dried in the sun and then mechanically ground, sieved to obtain homogeneous samples and finally packed in vacuum-sealed polyethylene bags and stored at room temperature until use.

### 3.2. Headspace Solid-Phase Microextraction (HS-SPME) Sampling

The HS-SPME sampling technique was used to collect the volatile components emitted by the dry pepper powders. In detail, approximately 2 mg of Baklouti and Paprika peppers was placed into a 4 mL glass vial with polytetrafluoroethylene (PTFE)-coated silicone septum. A SPME device from Supelco (Bellefonte, PA, USA), equipped with 1 cm fiber coated with 50/30 μm DVB/CAR/PDMS (divi-nylbenzene/carboxen/polydimethylsiloxane), was used to extract and adsorb the volatiles [55,56]. Before each single sampling, the fiber was conditioned at 270 °C for 30 min in order to remove any residual trace of the previous sampling. After the thermostating phase, the fiber was exposed to the headspace of the sample for 30 min at 50 °C. Lastly, SPME fiber was inserted in an injection port at 250° in splitless mode for the desorption of captured analytes.

### 3.3. GC-MS Analysis

The analyses were carried out using a gas chromatograph coupled to a mass spectrometer, the Clarus 500 model Perkin Elmer (Waltham, MA, USA), equipped with an FID (flame ionization detector). For the chromatographic separation, a Varian Factor Four VF-5 capillary column was used. The temperature program started from 50 °C then rose to 220 °C at 6°/min and was finally maintained at this temperature for 15 min. Hyper-pure helium was the carrier gas used at a constant rate of 1 mL/min. MS detection was performed with electron ionization (EI) at 70 eV operating in the full-scan acquisition mode in the *m*/*z* range of 40–550 amu, and the temperatures of the ion source and connecting parts were 180 °C and 200 °C, respectively. The identification of the volatile compounds was performed first through the comparison of the mass spectra with those stored in the Wiley 2.2 and Nist 11 mass spectral library databases; further, linear retention indices (LRIs), using a series of alkane standards (C_8_–C_25_), were calculated and compared with those available in the literature. The relative amounts of the components were expressed as percent peak area of the FID signal relative to total peak area without the use of an internal standard and any factor correction. The analysis was carried out in triplicate.

### 3.4. Proton Transfer Reaction–Time-of-Flight–Mass Spectrometry (PTR-ToF-MS) Analysis

Headspace analysis was conducted on samples following the setup and instrumental conditions outlined by Taiti et al. [26]. Each sample consisted of 10 g of dried spice and underwent analysis three times, with the average value calculated. VOC analysis was performed using a PTR-ToF-MS (8000 model, Ionicon Analytik GmbH, Innsbruck, Austria) with H_3_O^+^ as the reagent ion and within a mass range of *m*/*z* 20–220. In brief, each sample was individually sealed within an airtight jar and subjected to a 120 s analysis at a flow rate of 50 sccm. To mitigate the risk of contamination, the jars were thoroughly cleaned with purified air from a Zero Air Generator (Peak Scientific, Glasgow, UK) before starting the measurements. In the same way, the PTR inlet was consistently refreshed to ensure optimal conditions during the measurements. Moreover, noise thresholds were applied to refine the results. Specifically, peaks not significantly distinct from blank samples, signals associated with isotope mass peaks, peaks related to water chemistry (such as hydronium ions and water clusters), and other interfering ions (e.g., oxygen, nitrogen monoxide) were eliminated. Additionally, signals corresponding to ions present in trace amounts (<0.5 ppbv) were excluded.

### 3.5. GC-MS Analysis of the Spices After Derivatization

For the investigation of the non-volatile profiles of the spices, a derivatization reaction was performed [57]. To this aim, about 0.5 mg of each spice was added to 300 µL of pyridine and 100 µL of bis-(trimethylsilyl) trifluoroacetamide (BSTFA) with heating at 80 °C for 60 min. In total, 1 μL of the silylated sample was manually injected at 300 °C into the GC injector. The analysis was performed using the GC-MS apparatus and the same capillary column (Varian Factor Four VF-5) described in the previous section.

The GC oven temperature program was as follows: 80 °C followed by a gradient of 7 °C/min to 170 °C, then a gradient of 8 °C/min to 250 °C, and finally a gradient of 8 °C/min to 300 °C for 25 min. The identification of compounds was based on the percentage of similarity plus comparison of mass spectra (MS) using the NIST 11 data library.

### 3.6. Sample Preparation for Biological Tests

Two grams of each dry powder, Baklouti and Paprika, was introduced into graduated flasks containing 100 mL of 95% ethanol (*v*/*v*). Afterwards, the solutions underwent sonication for a duration of 30 min at a frequency of 50Hz (Starsonic 90 Digit, Liarre, Asti, Italy) at room temperature and, subsequently, it was filtered using counter paper. The resulting extract was then concentrated using a vacuum rotatory evaporator at a temperature of 40 °C (Rotavapor, Steroglass S.r.l, Perugia, Italy). To achieve stock concentrations of 100 mg/mL, the Baklouti and Paprika extracts were dissolved in dimethyl sulfoxide (DMSO, Sigma Aldrich, Milan, Italy), as indicated by Loizzo et al. [43].

### 3.7. GC-MS Analysis of Derivatized Extracts

To describe the chemical content of the extracts used for the biological essays, approximately 0.5 mL of the Baklouti and Paprika extracts was dried under a stream of nitrogen and then 300 µL of pyridine and 100 µL of bis-(trimethylsilyl) trifluoroacetamide (BSTFA) were added with heating at 80 °C for 60 min [58]. The derivatization reaction was performed inside a closed vessel. After 60 min of reaction, the sample was allowed to cool in a refrigerator at 4 °C. In total, 1 μL of each silylated extract was manually injected at 300 °C into the GC injector in split mode (1:20). The analyses were carried out using the same apparatus, the GC-FID/GC-MS. The oven temperature program was as follows: 70 °C followed by a gradient of 6 °C/minute to 170 °C, then a gradient of 7 °C/minto 250 °C, and finally a gradient of 8 °C/min to 300 °C for 25 min. Mass spectra were acquired in electron ionization mode. Identification and quantification were performed as reported in Section 3.5. The analysis was repeated in triplicate.

### 3.8. Bacterial Strains

In this study, eleven strains from various species and origins (as detailed in Table 6) were chosen to evaluate the antimicrobial efficacy. These bacterial strains belonged to the collection of the Department of Bioscience and Technology for Food, Agriculture, and Environment, University of Teramo (Italy). The cultures were grown on Müeller–Hinton (MH) agar plates (Liofilchem, Roseto degli Abruzzi, Italy); then, they were inoculated in Müeller–Hinton broth (Liofilchem) and incubated at 37 °C for 18 h, resulting in the formation of a fresh culture in the early stationary phase. Moreover, the fresh culture of *Campylobacter coli* was cultivated on CCDA (Liofilchem, Roseto degli Abruzzi, Italy) agar at 40 °C for 18 h utilizing the CampyGen™ kit (Thermo Scientific™, Milan, Italy). The standardized inocula were subsequently obtained by measuring the O.D 600 nm of the bacterial cultures in a Lambda bio 20 spectrophotometer and then suitably diluting them to a concentration of 10^7^ UFC/mL.

### 3.9. Bacterial Minimum Inhibitory Concentration (MIC) and Minimum Bactericidal Concentration (MBC) Assay

The determination of Baklouti and Paprika extracts was performed by the microdilution method, according to CLSI guidelines (2020) [59]. The negative control was assessed in the presence of DMSO alone. The determination of MIC values was performed at a temperature of 37 °C for all strains, and at 40 °C for *C. coli* by using a CampyGen™ kit, after 24 and 48 h. The MICs were defined as the lowest concentration of the solution where the absence of red discoloration of TTC (2,3,5-triphenyltetrazolium chloride, Sigma-Aldrich, Milan, Italy), previously added to MH broth (1 μL/mL), was observed. The MBCs were evaluated by striking a loop of inoculum onto MH agar plates, from the microplate wells of the MIC determination, after 24 and 48 h of incubation. The MBC was interpreted as the extract concentration determining no growth on the plates. The experimental analyses were carried out in three biological replicates.

### 3.10. Statistical Analysis

Statistical analyses were performed with GraphPad Prism 6.1 software. The experiments were replicated three times unless otherwise indicated, and the data were expressed as the average ± standard deviation (SD).

## 4. Conclusions

In the present work, the obtained results from the chemical analyses demonstrated that the characteristic flavor and aroma of two *Capsicum annuum* accessions (“Paprika” and “Baklouti”) are related to the specific type and combination of VOCs found. Both samples showed a higher amount of sesquiterpenes than monoterpenes, while only Baklouti pepper contained sugars and also emitted a higher number of sulfur-based compounds. Oleic acid was the main fatty acid detected in both peppers. The demonstrated antibacterial efficacy of the extracts, particularly against Gram-positive bacteria, is undoubtedly linked to the pool of detected metabolites. In summary, the findings may contribute to better a understanding of the metabolome of Paprika and Baklouti peppers collected in Tunisia in order to create new research perspectives for future applications in various sectors, from food to pharmaceutical, considering these matrices as sources of bioactive molecules.

## Figures and Tables

**Table 1:** Chemical composition (percentage mean value ± standard deviation) of Paprika and Baklouti peppers, as determined by SPME-GC/MS.

N°	Component ^1^	LRI ^2^	LRI ^3^	Paprika (%)	Baklouti (%)
1	*α*-pinene	935	937	0.6 ± 0.03	0.3 ± 0.02
2	limonene	1025	1028	8.6 ± 0.07	3.5 ± 0.04
3	linalool	1081	1088	6.6 ± 0.04	2.2 ± 0.05
4	camphor	1141	1139	0.9 ± 0.03	-
5	estragole	1190	1177	2.4 ± 0.05	1.2 ± 0.03
6	(-)-carvone	1238	1243	11.9 ± 0.10	6.0 ± 0.02
7	anethole	1255	1260	-	0.1 ± 0.00
8	isobornyl acetate	1270	1268	-	0.3 ± 0.03
9	*α*-limonene diepoxide	1297	1294	0.1 ± 0.01	-
10	carvacrol	1299	1297	-	1.0 ± 0.04
11	eugenol	1349	1345	-	0.2 ± 0.02
12	*δ*-elemene	1352	1347	0.3 ± 0.02	0.1 ± 0.01
13	cyclosativene	1370	*	0.2 ± 0.02	0.3 ± 0.02
14	*α*-copaene	1396	1393	4.1 ± 0.04	2.4 ± 0.03
15	*β*-elemene	1398	1396	1.2 ± 0.02	0.7 ± 0.02
16	*β*-copaene	1425	1427	-	0.1 ± 0.01
17	*trans*-*α*-bergamotene	1430	1432	1.0 ± 0.02	0.2 ± 0.02
18	*trans*-geranylacetone	1436	1434	-	0.3 ± 0.02
19	*β*-caryophyllene	1442	1440	13.0 ± 0.11	5.1 ± 0.04
20	*trans*-*β*-farnesene	1444	1441	0.5 ± 0.03	-
21	*β*-himachalene	1450	1447	1.8 ± 0.05	1.2 ± 0.03
22	*α*-curcumene	1479	1475	16.0 ± 0.09	19.7 ± 0.12
23	I-zingiberene	1492	1490	1.8 ± 0.03	25.5 ± 0.15
24	α-farnesene	1510	1506	1.5 ± 0.02	3.5 ± 0.02
25	*β*-bisabolene	1515	1512	10.5 ± 0.12	10.7 ± 0.11
26	*trans*-calamenene	1519	1515	-	0.3 ± 0.02
27	*β*-sesquiphellandrene	1520	1518	14.7 ± 0.15	13.8 ± 0.15
28	*α*-calacorene	1525	1528	-	0.1 ± 0.01
29	myristic acid	1761	1759	0.4 ± 0.02	0.2 ± 0.02
30	palmitic acid	1955	1951	0.9 ± 0.03	0.6 ± 0.02
31	oleic acid	2138	2141	1.0 ± 0.04	0.4 ± 0.02
	SUM			100.0	100.0
	Monoterpenes			27.9	13.9
	Sesquiterpenes			65.4	83.0
	Fatty acids			2.3	1.2
	Others			4.4	1.9

^1^ The components are reported according to their elution order on an apolar column; ^2^ linear retention indices measured on an apolar column; ^3^ linear retention indices from the literature; * LRI not available; -: Not detected.

**Table 2:** Chemical composition (percentage mean values) of spices, as determined by PTR-ToF-MS.

Peak Number	*m/z*	Chemical Formula	Tentative Identification	Paprika			Baklouti		
				Average	SD	% of the Total	Average	SD	% of the Total
1	27.022	C_2_H_3_^+^	Fragment	19.39	7.76	0.37	20.61	2.92	0.51
2	31.018	CH_3_O^+^	Formaldehyde	48.62	13.29	0.93	28.92	5.10	0.71
3	33.033	CH_5_O^+^	Methanol	1499.01	65.05	28.57	621.31	65.76	15.26
4	41.038	C_3_H_5_^+^	Alkyl fragment (alcohols and esters)	48.45	8.81	0.92	31.78	6.41	0.78
5	43.018	C2H3O^+^	Alkyl fragments	1144.25	176.78	21.81	1022.25	46.67	25.11
6	43.054	C_3_H_7_^+^	Alkyl fragment (alcohols)	56.01	16.24	1.07	58.00	11.95	1.42
7	45.033	C_2_H_5_O^+^	Acetaldehyde	180.77	44.05	3.45	134.97	44.90	3.32
8	47.013	CH_3_O_2_^+^	Formic Acid/Formates	224.78	38.54	4.28	135.73	22.06	3.33
9	49.011	CH_5_S^+^	S compound (Methanethiol)	2.60	1.40	0.05	0.00	0.00	0.00
10	55.054	C_4_H_7_^+^	C4 aldehyde fragments	69.97	5.11	1.33	105.41	17.48	2.59
11	57.033	C_3_H_4_O^+^	C3 aldehyde and ketone fragments	16.16	5.35	0.31	13.09	2.72	0.32
12	57.069	C_4_H_9_^+^	Alcohol fragments	9.31	3.10	0.18	14.72	1.36	0.36
13	59.049	C_3_H_7_O^+^	Propanal, acetone	61.13	18.03	1.17	143.83	39.95	3.53
14	61.028	C_2_H_5_O_2_^+^	Acetates	1139.07	377.60	21.71	1194.57	272.24	29.34
15	63.027	C_2_H_7_S^+^	Dimethylsulfide	10.03	0.37	0.19	6.84	0.31	0.17
16	67.054	C_5_H_7_^+^	Terpene fragment	4.59	0.01	0.09	4.49	0.12	0.11
17	69.033	C_4_H_5_O^+^	Furan	4.94	1.89	0.09	0.00	0.00	0.00
18	69.069	C_5_H_9_^+^	Isoprene/alkyl fragment (e.g., 2-methylbutanal, 1-octen-3-ol)	10.91	0.83	0.21	8.47	0.70	0.21
19	71.049	C_4_H_7_O^+^	2-Butenal	17.69	0.80	0.34	16.57	0.55	0.41
20	71.086	C_5_H_11_^+^	Alcohol (3-methyl-1-butanol, pentanol, iso-pentanol, 2-ethyl-1-hexanol, octanol, nonanol)	4.59	1.22	0.09	0.00	0.00	0.00
21	73.028	C_4_H_9_O^+^	Alkyl fragment	36.08	1.00	0.69	40.92	1.77	1.01
22	73.065	C_3_H_5_O_2_^+^	Isobutanal/butanone/butanal	325.25	34.65	6.20	235.10	59.33	5.77
23	75.044	C_3_H_7_O_2_^+^	Butanol/methyl acetate/propanoates	14.59	3.59	0.28	12.86	1.72	0.32
24	81.068	C_6_H_9_^+^	Terpene fragment/aldehyde fragment (trans-2-hexenal)	35.56	5.65	0.68	37.49	3.83	0.92
25	83.049	C_5_H_7_O_2_^+^	2-Methylfuran	3.48	1.22	0.07	3.87	2.19	0.09
26	83.086	C_6_H_11_^+^	C6 compounds/Hexenol fragment	3.26	0.80	0.06	4.10	0.29	0.10
27	85.065	C_5_H_9_O^+^	Methyl-butenal/1-penten-3-one	4.82	1.22	0.09	6.10	1.35	0.15
28	87.045	C_4_H_7_O_2_^+^	2,3-butanedione/diacetyl	26.21	6.84	0.50	21.45	4.58	0.53
29	89.059	C_4_H_9_O_2_^+^	Ethyl acetate	12.93	0.53	0.25	14.44	0.66	0.35
30	93.069	C_7_H_9_^+^	Toluene/terpene fragments (e.g., cymene, limonene)	4.23	0.22	0.08	1.41	0.73	0.03
31	95.049	C_6_H_7_O^+^	Phenol	4.61	0.58	0.09	3.83	0.11	0.09
32	95.086	C_7_H_11_^+^	1-Methyl-1,4-cyclohexadiene/terpene fragments	7.25	3.07	0.14	8.11	0.07	0.20
33	97.064	C_6_H_9_O^+^	2-Ethylfuran	8.25	4.41	0.16	2.94	0.05	0.07
34	103.075	C_5_H_11_O_2_^+^	Ethyl 3-methylbutanoate/3-methylbutanoic acid	6.80	0.45	0.13	7.77	0.22	0.19
35	105.069	C_8_H_9_^+^	Styrol	5.62	1.55	0.11	4.50	1.48	0.11
36	107.049	C7H7O^+^	Benzaldehyde	6.89	0.64	0.13	1.84	0.57	0.05
37	107.085	C_8_H_11_^+^	p-Xylene	3.99	1.21	0.08	6.38	0.07	0.16
38	109.064	C_7_H_9_O^+^		39.24	8.49	0.75	19.16	3.31	0.47
39	109.101	C_8_H_13_^+^	Terpenes fragments	27.61	9.91	0.53	12.92	4.89	0.32
40	111.038	C_3_H_11_O_2_S^+^	S+ compound	3.98	2.06	0.08	0.00	0.00	0.00
41	119.085	C_9_H_11_^+^	Terpene fragment	5.34	1.39	0.10	2.63	1.33	0.06
42	121.101	C_9_H_13_^+^	Terpene fragment	3.87	0.59	0.07	1.89	0.75	0.05
43	123.117	C_9_H_15_^+^	Sesquiterpene fragments	18.67	4.01	0.36	10.30	2.30	0.25
44	135.117	C_10_H_15_^+^	p-Cymene/monoterpene ketone fragment	3.99	2.10	0.08	2.38	0.10	0.06
45	137.132	C_10_H_17_^+^	Monoterpenes (e.g., limonene)	9.63	5.33	0.18	12.68	3.08	0.31
46	139.111	C_9_H_15_O^+^	Nonadienal/2-pentyl-furan	3.87	2.99	0.07	2.37	0.55	0.06
47	149.132	C_11_H_17_^+^	Sesquiterpene fragments or ectocarpene hydrocarbons	5.09	2.15	0.10	4.56	0.80	0.11
48	153.126	C_10_H_17_O^+^	Terpenoid like-compounds (e.g., β-cyclocitral)/(E,E)-2,4-decadienal	5.06	2.08	0.10	4.30	0.10	0.11
49	205.195	C_15_H_25_^+^	Sesquiterpenes	15.68	4.15	0.34	17.51	3.08	0.18
**Total Emission intensity**				5224.07		100.00	4065.37		100.00

**Table 3:** Chemical composition (percentage mean values ± standard deviation) of spices after derivatization as determined by GC/MS.

N°	COMPONENT	Paprika (%)	Baklouti (%)
1	oleic acid (18:1)	47.3 ± 0.25	9.1 ± 0.08
2	palmitic acid (16:0)	17.7 ± 0.10	4.6 ± 0.04
3	linoleic acid (18:2)	28.1 ± 0.19	4.7 ± 0.05
4	stearic acid (18:0)	4.3 ± 0.03	0.5 ± 0.02
5	arachidic acid (20:0)	2.6 ± 0.02	3.3 ± 0.03
6	D-galactopyranose	-	0.8 ± 0.02
7	D-mannitol	-	1.8 ± 0.03
8	D-psicofuranose	-	10.5 ± 0.12
9	D-ribofuranose	-	4.2 ± 0.03
10	D-fructofuranose	-	4.7 ± 0.05
11	lyxose	-	1.6 ± 0.03
12	glycerol	-	54.2 ± 5.50
	SUM	100.0	100.0
	Fatty Acids	100.0	22.2
	Sugars		23.6
	Sugar alcohols		54.2

- Not detected.

**Table 4:** Chemical composition (percentage mean values ± standard deviation) of the extracts after derivatization as determined by GC/MS.

N°	Component	Paprika (%)	Baklouti (%)
1	dimethyl sulfone	6.0 ± 0.07	4.9 ± 0.06
2	mercaptoethanol	-	0.6 ± 0.02
3	1,4-dithiane	3.0 ± 0.03	1.4 ± 0.04
4	benzothiazole	2.2 ± 0.04	tr
5	ethene, 1,2-bis(methylthio)-	3.5 ± 0.05	3.1 ± 0.05
6	2-phenylethanethiol	0.5 ± 0.03	0.2 ± 0.02
7	disulfide, methyl(methylthio)methyl-	2.4 ± 0.02	2.2 ± 0.07
8	2,3-butanediol	-	0.7 ± 0.03
9	glycerol	-	4.9 ± 0.08
10	glyceric acid	-	0.5 ± 0.02
11	tagatofuranose	-	0.4 ± 0.02
12	sorbofuranose	-	0.9 ± 0.05
13	D-fructofuranose	-	2.4 ± 0.06
14	D-mannitol	-	0.7 ± 0.02
15	D-sorbitol	-	2.1 ± 0.06
16	galactopyranose	-	1.4 ± 0.04
17	palmitic acid	22.7 ± 1.82	10.0 ± 0.80
18	linoleic acid	12.3 ± 0.92	9.0 ± 0.70
19	oleic acid	46.8 ± 3.11	54.5 ± 5.30
	SUM	99.5	99.9
	Sulfur Compounds	17.6	12.4
	Fatty Acids	81.8	73.5
	Sugars	-	5.1
	Sugar acids	-	0.5
	Sugar alcohols	-	7.7
	Others	-	0.7

tr: percentage mean value < 0.1%; -: not detected.

**Table 5:** MICs and MBCs after 24 and 48 h of Baklouti and Paprika extracts against the bacterial strains under study.

	Baklouti				Paprika			
Bacterial Strains	MIC		MBC		MIC		MBC	
	24 h	48 h	24 h	48 h	24 h	48 h	24 h	48 h
*C. coli* ATCC 11353	12.5	12.5	12.5	12.5	12.5	12.5	12.5	12.5
*E. coli* ATCC 35218	12.5	12.5	12.5	12.5	12.5	12.5	12.5	12.5
*L. monocytogenes* ATCC 7644	12.5	25	25	25	12.5	25	25	25
*L. monocytogenes* 641/6II	25	25	25	25	25	25	25	25
*L. monocytogenes* 13155/2017	25	25	25	25	25	25	25	25
*S.* Enteritidis ATCC 13076	12.5	12.5	12.5	12.5	12.5	12.5	12.5	12.5
*S.* Veneziana 2	˃50	˃50	˃50	˃50	50	50	˃50	˃50
*S.* Enteritidis 1	˃50	˃50	˃50	˃50	50	50	˃50	˃50
*S. aureus* ATCC 43300	0.78	0.78	0.78	0.78	<0.39	<0.39	<0.39	<0.39
*S. aureus* aA 17	25	25	25	25	25	25	25	25
*S. aureus* aA 19	25	25	25	25	25	25	25	25

MIC and MBC values are expressed as mg/mL and the standard deviation is not reported, being zero for all replicates. MICs, Minimum Inhibitory Concentrations; MBCs, Minimum Bactericidal Concentrations.

**Table 6:** Overview on the bacterial strains under study.

ID Code	Strains	Origin
ATCC 11353	*Campylobacter coli*	Type strain
ATCC 35218	*Escherichia coli*	Type strain
ATCC 7644	*Listeria monocytogenes*	Type strain
ATCC 13076	*Salmonella enterica* subsp. *enterica* serovar Enteritidis	Type strain
ATCC 43300	*Staphylococcus aureus*	Type strain
641/6II	*Listeria monocytogenes*	Cold smoked salmon
13155/2017	*Listeria monocytogenes*	Dairy products
2	*Salmonella enterica* subsp. *enterica* serotype Veneziana	Fresh and minimally processed vegetables
1	*Salmonella enterica* subsp. *enterica* serovar Enteritidis	Tiramisù
aA 17	*Staphylococcus aureus*	Salami
aA 19	*Staphylococcus aureus*	Salami

## Data Availability

The data presented in this study are available on request from the corresponding author due to restrictions of privacy.
